# Operating theatre nurses’ self‐reported clinical competence in perioperative nursing: A mixed method study

**DOI:** 10.1002/nop2.352

**Published:** 2019-08-15

**Authors:** Ann‐Catrin Blomberg, Lillemor Lindwall, Birgitta Bisholt

**Affiliations:** ^1^ Department of Health Sciences Karlstad University Karlstad Sweden

**Keywords:** competence, mixed method, operating theatre nurse, perioperative nursing

## Abstract

**Aims:**

The aim of this study was to investigate how operating theatre nurses (OTNs) self‐rated their clinical competence and describe their experience of important factors for the development of clinical competence in perioperative nursing.

**Design:**

A cross‐sectional study with a mixed‐method approach was chosen. Data were collected through a modified version of the questionnaire *Professional Nurse Self‐Assessment Scale of Clinical Core Competence I,* which was supplemented with an open‐ended question.

**Methods:**

Data were collected from 303 operating theatre nurses in Sweden. Statistics analysis was used to identify the relationship between the participants' background variables. The open‐ended question was analysed by using a qualitative conventional content analysis.

**Results:**

Academic degree and professional experience of perioperative nursing were significant for the development of clinical competence. Academic degree appeared to affect operating theatre nurses’ leadership and cooperation in the surgical team, as well as how consultations took place with other professionals.

## 1 INTRODUCTION

High‐tech operating theatres today offer various challenges that require increasingly specialized and qualified operating theatre nurses (OTNs) who have had continuous development of clinical competence in perioperative nursing (Sweeney, 2010; Smith & Palesy, 2018). OTNs’ clinical competence must be constantly reassessed with regard to different perioperative situations, and OTNs have responsibility for ensuring good and safe nursing care, before, during and after surgery. Luck and Gillespie (2017) pointed out that competence for more advanced medical technology in perioperative nursing is requested. Sweeney (2010) expressed concern that increased focus and dependence on medical technology could reduce human contact with patients. Some studies have focused on the challenge faced by OTNs who must be a nurse as well as a skilled medical technician (Richardson‐Tench, 2008; McGarvey et al., 2000). According to Blegeberg et al. (2008), OTN participation in the patient's nursing process was incomplete. This means that OTNs’ clinical competence in perioperative nursing is unclear as well as their relationship with the patient. With this in mind, the goal was to use a questionnaire to investigate how OTNs self‐rated their clinical competence in perioperative nursing.

## 2 BACKGROUND

Perioperative nursing is described in this study, in line with Lindwall and von Post (2000) as: A nurse anaesthetist's and operating theatre nurse's pre‐, intra‐ and postoperative care for a patient who is undergoing surgery. Perioperative nursing includes all nursing activities related to the surgical treatment, organization and leadership of perioperative practice. Perioperative dialogues are a nurse anaesthetist's and operating theatre nurse's pre‐, intra‐ and postoperative dialogues with the patient, with the purpose of planning, implementing and evaluating perioperative nursing and creating continuity in patient care (Blomberg et al. 2018a). Within perioperative nursing, professional OTNs are required to have specific clinical competence to be able to work independently and to be able to cooperate in a surgical team in order to ensure patient safety.

The International Council of Nurses (ICN) (2013) emphasizes that the profession is based on an ethical responsibility to safeguard the patient's interest, to show humility and respect, as well as to promote the patient's right to self‐determination. The OTNs’ profession is governed by an ethical code, a commitment and a critical approach to what is right and wrong. It includes having the autonomy to be able to make decisions on your own and to stand for their consequences. Being a professional means having the knowledge, skills and abilities as well as being able to apply research results, “knowing that” and “knowing how” a specific task should be done and why, as well as having personal responsibility for what is right and wrong in the situation (Bentling & Jonsson, 2010). There are several definitions of the concept competence and the meaning varies. According to Ellström (1992), competence means having the ability to act and perform the specifics of duty in a certain situation or context, and to reflect on and critically analyse and evaluate one's own way of carrying out the work. Further, professional competence is defined as the nurse's capacity to act and to integrate knowledge, skills, attitudes and values in different healthcare contexts (Cowin et al., 2008; Meretoja, Isoaho, Isoaho, & Leiono‐Kilpi, 2004). However, the concept of competence varies within different professions and in different contexts.

Clinical competence is not just about education and experience but the ability to unite theory and practice. The Aristotelian view of knowledge involves acting wisely based on practical wisdom and ethical attitude, being able to choose what is good or bad for a person in a situation. It can be described as “knowledge in action”, based on Aristoteles (2012). Development of clinical competence is a prerequisite for being able to make responsible decisions in relation to the patient's care (Meretoja, Isoaho, et al., 2004). According to Eriksson and Lindström (2003), clinical competence takes place in nursing activities in the situation and refers to concrete caring in situations where the patient and caregivers cooperate towards the “patient bed.” Bull and FitzGerald (2004, 2006) believe that OTNs have the competence to combine medical technology knowledge with caring, but that it is not always visible in a high‐tech environment. According to Smith and Palesy (2018), this can entail prioritizing developing competence in medical technology instead of competence in nursing care within perioperative nursing.

Previous research (Boyle, Cramer, Potter, Gatua, & Stobinski, 2014; Bull & FitzGerald, 2006; Gillespie & Hamlin, 2009; Stobinski, 2015) has asked for research to clarify clinical competence in the relationship between nursing actions and caring for the patient because it is not yet fully understood in perioperative nursing. Meretoja, Leino‐Kilpi, and Kaira (2004) compared nurses’ professional competence in intensive care, emergency care and theatre care, and the result showed that OTNs rated their competence higher in managing situations, but lower in diagnostic functions and teaching/coaching, because of lacking relationships with patients. To identify the underlying dimensions of OTNs’ competence in perioperative nursing, the Perceived Perioperative Competence Scale‐Revised (PPCS‐R) was used and showed differences related to professional experience and education (Gillespie, Chaboyer, Wallis, & Werder, 2011; Gillespie & Hamlin, 2009; Gillespie, Polit, & Chaboyer, 2013). Jaensson, Falk‐Brynhildsen, Gillespie, Wallentin, and Nilsson (2018) tested the PPRC‐S in a Swedish context and showed that OTNs rated their competence lower in the factor of empathy, which was about a caring relationship with the patient. Finally, the instrument has been used to examine OTNs’ knowledge and skills, but there is limited research on the use of a questionnaire to study self‐rated OTNs’ clinical competence in perioperative nursing care. OTNs’ clinical competence as well as their formal and personal responsibility in the care of the patient has been shown in previous research to be partly invisible (Blomberg et  al. 2018a; Blomberg, Bisholt, Nilsson, & Lindwall, 2014). Our research question is how OTNs perceive their clinical competence and what influences the development of clinical competence in perioperative nursing? *The aim* of this study was to investigate how OTNs self‐rated their clinical competence and to describe their experience of important factors for the development of clinical competence in perioperative nursing.

## 3 THE STUDY

### 3.1 Design and setting

A cross‐sectional study design with a mixed method approach according to convergent parallel design (Creswell, 2014) was conducted in 2016. A core assumption of mixed method is that when quantitative and qualitative data are combined, the collective strength provides a better understanding of the research question than if one data source had been used. Mixed methods research is defined by Creswell (2014) as “*research in which the investigator gathers both quantitate (close‐ended) and qualitative (open‐ended) data, integrates the two, and then draws interpretation based on the strengths of both sets of data to understand the research problem*”(Creswell, 2014, p. 2). Collection of the quantitative and qualitative data was simultaneous, and the presentation of findings from the different sources can be integrated into the result or discussion sections. The results are integrated into this study in the discussion section.

Sweden is divided into six medical care regions, which organize highly specialized care based on need and availability. These comprise several regions/counties and at least one university hospital respectively, which functions as a research and teaching centre, as well as performing advanced care. Several districts comprise a region/county with a large regional/central hospital with various general surgical specialties. Each region/county has several district hospitals with fewer surgical specialties. OTNs’ education has changed over the years, which means that in perioperative practice there are OTNs with a postgraduate education and direct education without nursing as a subject, something which can later be supplemented to attain a registered nurse (RN) degree. When the RN education programme was lengthened to three years (corresponding to 180 credits in the European Credit Transfer System), an academic bachelor's degree was introduced. In accordance with the Bologna process, the postgraduate education in theatre care is carried out at an advanced level in Sweden**.**

### 3.2 Participants

In total, 1,057 OTNs were asked to participate, and 303 answered (response rate 28%). Inclusion criteria were clinically active OTNs working in operating theatres in Sweden. The participants were OTNs at universities or central/regional and district hospitals, who worked in different surgical specialities (Table 2).

### 3.3 Instruments and measurement

The modified questionnaire Professional Nurse Self‐Assessment Scale of Clinical Core Competence I (PROFFSNurse SAS I) was chosen to investigate OTNs’ self‐rated clinical competence in perioperative nursing, because it focuses on dynamic and mutual nurse–patient relationships. Psychometric testing has been carried out and shown to be acceptable and reliable to construct validity in the assessment of practicing clinical competence in long‐term and home care contexts in Norway (Finnbakk, Wangensteen, Skovdahl, & Fagerström, 2015). The PROFFSNurse SAS I language was Swedish. Two items were added from the Nurse Clinical Competence Scale (NCCS) questionnaire that involved implementing research and reporting incidents to ensure patient safety (Finnbakk et al., 2015). To ensure face validity, the research group (ACB, BB, and LL) assessed the clarity, wording, understanding and relevance of the questionnaire in perioperative nursing. A pilot test was then conducted with 10 OTNs from different operating theatres in Sweden, to test the applicability of the questions, which resulted in 10 items from the PROFFSNurse SAS I questionnaire being revised. Excluded items included clinical assessment of the patient's diagnosis and counselling on prevention and rehabilitation, which were not relevant in perioperative nursing. The questionnaire was then reviewed. Approval of the revision was performed by the research group who developed the original questionnaire (Finnbakk et al., 2015) for use in perioperative nursing. The modified version of PROFFSNurse SAS I contained 43 items across six components, and the number of items in each component and Cronbach alpha for this study is shown in Table 1. The numeric options for the numeric rating scale were enclosed in ten boxes, and the scale ranged from 1 to 10, where 1 indicated a lack of clinical competence and 10 full clinical competence. Each item was asked in reference to self‐assessment of clinical competence and the need for further education. Only results from the OTNs’ self‐assessment of clinical competence are presented in this study, and not those from the need for further education.

**Table 1:** Descriptive statistic for the six components of the modified PROFFSNurse SAS I (*N* = 303)

PROFFSNurse SAS components	Number of items	Mean	*SD*	Observed range	Cronbach´s α
Direct clinical practice	15	109.4	17.4	42–150	0.87
Professional development	5	37.3	7.6	16–50	0.79
Ethical decision‐making	10	74.8	12.4	38–100	0.84
Clinical leadership	4	35.1	3.8	20–40	0.74
Cooperation and consultation	6	51.4	5.6	34–60	0.67
Critical thinking	3	23.1	4.3	4–30	0.71

### 3.4 Data collection

Data were collected digitally from the modified questionnaire PROFFSNurse SAS I in perioperative nursing. The questionnaire was supplemented with an open‐ended question to describe factors of importance for the development of operation theatre nurses’ clinical competence. An inquiry about study participation was made to all head of departments in all 21 regions/counties in Sweden. One county did not reply and was therefore excluded. After study approval, the head of department was asked to forward our inquiry to a contact person to gain access to email addresses for possible participants. When the participants answered, they also gave their informed consent to participate. Four reminders were sent at 14‐day intervals.

### 3.5 Data analysis

The quantitative data were analysed using descriptive statistics to describe the participants’ background such as age, educational background, academic degree, place of employment and professional experience in perioperative nursing and from various surgical specialities (Table 2). The choice of these factors was discussed in the research group, and it was considered interesting to investigate whether it affected the development of clinical competence. The participants' background factors were classified into variables, and the answers were given on an ordinal scale. The participants rated themselves on a numeric scale 1–10. Analytical statistics were performed by analysis of variance (ANOVA) to search for relationships between the OTNs’ background factors and the six components of the modified PROFFSNurse SAS I. Each component was used in the self‐assessment as a dependent variable. The continuous variable age was used as a covariate variable. When statistically significant data emerged related to the participants' background factors, a post hoc test was conducted to investigate which group differed from the others in each component. The level of statistical significance was set to α = .05. Data were analysed using the Statistical Package for the Social Sciences (SPSS), version 25.

**Table 2:** Demographic background of operating theatre nurses’ (OTN) in the sample (*N* = 303)

Demographics characteristics	*N* (%)
Gender	
Female	287 (95)
Male (Missing 2)	14 (5)
Age (years)	
Mean score (*SD*)	46 (9.5)
Range (Missing 8)	26 – 65
Educational background	
2‐year direct education and no postgraduated in theatre care	92 (30.4)
RN education with 1‐year postgraduate in theatre care	70 (23.1)
RN education with 1‐year advanced nursing in theatre care (Missing 2)	139 (45.9)
Academic degree	
Bachelor degree	78 (26.2)
Master degree (60 credits)^a^	96 (32.2)
No academic degree (Missing 5)	124 (41.6)
Professional experience	
<10 years	136 (45.2)
10 years–20 years	64 (21.3)
>20 years (Missing 2)	101 (33.6)
Place of employment	
University hospital	100 (33.4)
Central/Regional hospital	95 (31.8)
District hospital (Missing 4)	104 (34.8)
Professional experience in various surgical specialties^b^	
General surgery (Urology, Gynaecology, Vascular)	262 (86.5)
Orthopaedic ( Hand)	216 (71.3)
Thoracic	38 (12.5)
Plastic	106 (35)
Neurosurgery	35 (11.6)
Ear–nose–throat	142 (47)
Eye	19 (6)

Abbreviation: RN, Registered Nurse.

a Master degree (60 credits) corresponds to one year at Master programme.

b Participants were able to select multiple options.

A qualitative conventional content analysis (Hsieh & Shannon, 2005) was performed of the responses offered by the participants from the open‐ended question. The responses were read several times to get an overall sense of the content, then read more closely to identify meaning units that described OTNs’ perceptions of factors of importance for the development of clinical competence in perioperative nursing. The codes were then sorted, based on their similarities and differences, into categories and finally organized into themes.

### 3.6 Ethical considerations

The local university ethics committee approved the study (2015/722). Ethical standards of research were followed in accordance with the Declaration of Helsinki (2013). Participant confidentially was guaranteed. Participants had no face‐to‐face contact with the researchers, and the only contact was digital. When the questionnaire was sent out, an information letter was attached in which the participants were informed about the study's purpose and that participation was voluntary.

## 4 RESULTS

The results of each phase are presented separately with a synthesis of findings presented in the discussion sections. The results of the descriptive analysis showed that 58% of participants had an academic degree (Table 2). The OTNs’ educational levels were as follows: direct education and no postgraduate education in theatre care 30%, RN education with postgraduate education in theatre care 23% and advanced nursing in theatre care 46%. Over half of the OTNs had more than 10 years of professional experience in perioperative practice. The distribution of the OTNs between universities, regional/central and district hospitals was about the same. OTNs often had experience from several surgical specialities, particularly if they were employed in central/regional and district hospitals. The mean and standard deviation and how OTNs rated their clinical competence in the six components are shown in Table 1.

### 4.1 Relationship in clinical competence between academic degree and professional experience

Academic degree was statistically significant in *clinical leadership, cooperation and consultation, direct clinical practice, professional development* and *ethical decision‐making* (Table 3). Participants with a bachelor's degree (α = .041) rated themselves higher in *clinical leadership* compared to participants with no academic degree. OTNs with a master's degree of 60 credits rated themselves the lowest. Regarding *cooperation and consultation,* OTNs with a bachelor's degree (α = .009) also rated themselves highest. However, OTNs with no academic degree rated themselves lower than the ones with a master's degree of 60 credits in *cooperation and consultation.* Participants with an academic degree rated themselves higher than the other participants in *direct clinical practice*. In *professional development,* participants with a bachelor's degree (α = .010) rated themselves higher than the other participants. It was also shown here that participants with a master's degree of 60 credits rated themselves higher in *professional development* than in *direct clinical practice.* In *ethical decision‐making,* OTNs with an academic degree rated themselves higher than participants with no academic degree.

**Table 3:** Factors of importance for the development of clinical competence in perioperative practice (*N* = 303 OTNs)

The six components of the PROFFSNurse SAS I	Clinical leadership			Cooperation and consultation			Direct clinical practice			Professional development			Ethical decision‐making			Critical thinking		
	*F*	β^b^	*p* a	F	β^b^	*p* a	F	β^b^	*p* a	F	β^b^	*p* a	F	β^b^	*p* a	F	β^b^	*p* a
	*R* ^2^ 0.144			*R* ^2^ 0.093			*R* ^2^ 0.141			*R* ^2^ 0.203			*R* ^2^ 0.052					
Demographic background (*N*)																		
Academic degree	3.973		0.020^a^	3.638		0.028^a^	4.866		0.009^a^	3.735		0.025^a^	3.623		0.028^a^			
Bachelor degree (78)	1.353^d^			2.677^d^			6.089			3.284^d^			5.338					
Master degree 1 year (96)	–0.205			1.227			0.143			1.252			6.001					
No academic degree (124)	0^c^			0^c^			0^c^			0^c^			0^c^					
Professional experience in perioperative nursing	7.373		0.001^a^	8.353		0.001^a^	4.985		0.008^a^	9.433		0.001^a^						
<10 years (136)	–1.860			−4.256			−11.002			–7,303								
10–20 years (64)	–2.390^d^			–3.292			−7.102			–4,640								
>20 years (101)	0^c^			0^c^			0^c^			0^c^								

a Significant level at *p*‐value > 0.05, measured by ANOVA analysis.

b Standardized regression coefficient.

c This parameter is set to zero because it is redundant.

d Fisher's LSD test.

When it came to professional experience from perioperative nursing, OTNs with more than 20 years’ experience rated themselves higher than participants with less experience in *cooperation and consultation, direct clinical practice* and *professional development* (Table 3)*.* OTNs with 10–20 years of professional experience (α = .026) rated themselves lower in *clinical leadership* than participants with less than 10 years of professional experience. In addition, OTNs with more than 20 years of professional experience rated themselves highest in *clinical leadership*. When it came to *critical thinking,* there was no statistically significant relationship to the OTNs background variables (Table 3).

The open‐ended question was answered by 66 (21%) of the 303 OTNs, where they described factors of importance for the development of clinical competence in perioperative nursing. The contents from the open‐ended question were brought together in two themes and four categories: Need for *competence development, interprofessional learning, current routines/habits and inadequate resources*. The results are presented in two themes: *Factors that promote development of clinical competence* and *factors that hinder the development of clinical competence.*

### 4.2 Factors that promote the development of clinical competence

Education programmes for OTNs were previously not scientifically oriented. But the OTNs themselves felt that an academic degree was necessary for their clinical competence development:

*My education as an operating theatre nurse was very focused on clinical skills and unfortunately not so much on scientific knowledge. This is something that I consider necessary to develop my competence.*

To develop clinical competence, both formal education and informal education are required. Previous experience as a RN was an advantage in the assessment of the patient's clinical condition. The OTNs also thought that professional experience in perioperative nursing with additional education in both medical technology and perioperative nursing was needed for the development of clinical competence and was not dependent on professional experience. It also emerged that it was more favourable for the development of clinical competence in smaller hospitals than university hospitals because university hospitals have requirements for higher production, meaning time for the preparation of each patient is limited. This applies even to patients requiring more care:

*… it is good for those who get an education now because they have…more general medical knowledge that is also beneficial to surgery. If you feel it's easier…to take a larger part of the patient and be more involved in clinical assessments…The knowledge I have today has come from experience and supplementary education. More development is needed even if you have worked for a long time.*

…*working as an OTN at a large university hospital, compared to a smaller unit means limited time for preparation per patient… in addition, the patients have different levels of sickness…*

Another important factor was interprofessional learning with ongoing meetings and discussions with colleagues, preferably with colleagues from other surgical specialities. An open environment to share and gain knowledge as well as being kept up to date was important:

*…Important to have meetings in the workplace with a section in focus, preferably with those who are not located there. There you can ask each other, give each other tips, discuss and decide…New medical devices, new advice about everything from surgical material to how, what and why we do things…*

### 4.3 Factors that hinder the development of clinical competence

OTNs express a desire to be more involved in patients’ care but are prevented by current routines and habits in perioperative practice. This causes difficulties in planning nursing via consultation with the patient that is based on the patient's problems and needs. It was mainly younger OTNs who thought that current routines hindered the development of their clinical competence and patient care:

*Sometimes work in the workplace is made so that it becomes difficult for OTNs to get enough of the patient, it is easy to become “the anaesthesia patient”*

*…to have time to develop their own way of working without input from older colleagues*

Based on current routines and habits, there was limited time for the development of clinical competence in perioperative nursing. OTNs emphasized that competence development in medical technology was prioritized. They thought more time should be assigned to competence development and that medical technical knowledge should be supplemented with perioperative nursing for the development of patient care:

… *limited time for competence development, especially in nursing care… Courses are instead given in suture technology, endoscopy, treatment of patients with specific diagnoses – i.e. the doctors´ duties. We are losing more ground within our "core subject" and it goes beyond the care that patients benefit from. This development is deeply worrying.*

Inadequate resources in the form of time, staffing, financial budget and a constant requirement for production as well as not being able to influence decisions already taken were factors that hindered the development of clinical competence. OTNs emphasized the limited opportunities to participate in education, organized nationally or at the workplace, or to participate in a wide range of quality and development work. Some OTNs pointed out that some colleagues chose to quit because the work no longer met their expectations about what good care is.

*In the pressured situation that prevails, it is difficult to keep up with development competence. There is no time for reflection, and it is hard to find enough time to go away on education and have different kinds of competence development in the workplace. Today it is production that counts. This is a problem for the long‐ term development of OTNs’ knowledge and competence.*

*Colleagues end up running around. This is not the job they were educated to do*

### 4.4 Discussion

The study investigates the relationship between the OTNS’ self‐rated clinical competence background factors and describes the factors of importance for the development of clinical competence in perioperative nursing. This study shows that OTNs’ academic degree and professional experience were the background factors that influenced how they self‐rated clinical competence in perioperative nursing. The result showed that an academic degree, professional experience and interprofessional learning are important for the development of clinical competence in perioperative nursing. OTNs with a bachelor's degree rated themselves high regarding *clinical leadership, cooperation and consultation and professional development* compared to OTNs with no academic degree. According to Aiken et al. (2014) and Gillespie, Chaboyer, and Wallis (2007), RNs with an academic degree are associated with improved patient outcomes, since they have a professional approach, communicate more efficiently and use problem‐solving skills in a more complex manner. A previous study by Gillespie et al. (2011) also showed that OTNs with an academic degree were associated with an increased situational awareness in leadership and cooperation within the surgical team. Blomberg et al. (2018a) show that OTNs had the ability to take responsibility, when problems arose related to medical judgement, patient positioning as well as hygiene and sterility for patient safety. According to Jerlock, Falk, and Severinsson (2003), an academic degree could produce independent and autonomous RNs with the ability to acquire, update and assimilate new knowledge in nursing. In this study, the open‐ended question showed that OTNs wanted to learn more about most recent research and to participate in quality and improvement work, because of this, they wanted more scientific knowledge.

Operating theatre nurses with an academic degree also rated themselves higher in *ethical decision‐making* than participants with no academic degree. In the study of Blomberg et al. (2018a), it emerged that the nursing care and responsibility of the OTNs are based on ethical values: seeing and listening to the patient as a unique person. No significant relationships were shown across the background variable regarding *critical thinking.* This indicates a professional approach and involves both a critical attitude and an ethical attitude. Blomberg, Bisholt, and Lindwall (2018b) show that ethical value conflicts arose in perioperative practice when OTNs were prevented from being present in the perioperative nursing process because of current habits.

A surprising result in the present study was that OTNs with a master's degree of 60 credits rated themselves lower regarding *clinical leadership* but higher in *cooperation and consultation* and *professional development* compared to those with no academic degree. The results also showed that OTNs with no academic degree requested scientific knowledge, but other OTNs claimed that scientific competence is not utilized. Ellström (1992) points out that a person can hold competences not required for a certain job, and therefore, perhaps the OTNs’ scientific competence is not utilized, which indicates a need for the employers to make better use of their clinical competence to improve patients’ care within perioperative nursing.

A relationship between academic degree and professional experience in perioperative nursing showed that OTNs with a bachelor's degree and those with no academic degree rated themselves higher regarding *clinical leadership* than those with a master's degree of 60 credits. Interestingly, OTNs with 10–20 years of professional experience rated themselves lower in *clinical leadership* than those with less than 10 years of professional experience. Maybe it is the OTNs with a master's degree who rated themselves higher in clinical leadership. Aiken et al. (2014) showed that patients suffer from fewer complications after surgery, when nurses with an academic degree were responsible for the care. The result also shows that OTNs with more than 20 years of professional experience rated themselves higher in cooperation within the surgical team and consultations about patient care with other professions in perioperative practice. It also emerged that OTNs with more than 20 years of professional experience rated themselves higher in *direct clinical practice* and *professional development.* More extensive professional experience leads to OTNs planning their work more efficiently regardless of the type of surgery, and according to Mitchell et al. (2011), they use “judicial wisdom” to interpret the body language of the surgeon to be “one step ahead,” as well as “situation awareness” to be able to act based on previous professional experience. This also emerged in Blomberg et al. (2018a). According to Aiken, Clarke, Cheung, Sloane, and Silber (2003), professional experience is not a substitute for an academic degree which also reflects the present study, where competence developed was not dependent on professional experience.

In this study, the OTNs thought that smaller hospitals developed their clinical competence more than university hospitals. This was because university hospitals have a higher requirement for production and the time for preparation with each patient is limited. Spruce (2013) states that the complexity of perioperative nursing in addition to the challenging situations and fast space may overshadow the importance of perioperative nursing care. However, Gillespie et al. (2007) consider it to be more beneficial for the development of competence to be active in a university hospital, since more research is carried out there and there are more opportunities to take part in quality and development projects.

Operating theatre nurses pointed out in the open question that previous professional experience as an RN was an advantage when assessing the clinical condition of patients. They had supplemented their competence in nursing through formal and informal education. The result is in accordance with Gillespie et al. (2011) who show that experience as an RN has significance in planning, conducting and evaluating patient care. Previous OTN education was more focused on clinical skills rather than nursing care for the patient, and in this study, the result showed that competence development in perioperative nursing focused more on medical technology, which worried some of the OTNs. The result also showed that OTNs requested competence development in perioperative nursing care after completing OTN education. According to Blomberg et al (2014, 2018a), the OTNs wanted to alleviate suffering and to ensure patient wellbeing. Stobinski (2015) states that the employer is the one who prioritizes the necessary competence development to keep up to date, but OTNs also have personal responsibility. This leads to limited possibilities for the development of patient care. In addition, OTNs wanted more time for interprofessional learning with colleagues working in other surgical specialties, something that was also brought up in a study by Gillespie, Chaboyer, Longbottom, and Wallis (2010).

### 4.5 Methodological limitations and strengths

The response rate in this study was 28%, which could be considered low. Holbrook, Krosnik, and Pfent (2007) highlighted that studies with a response rate even as low as 20% might offer just as precise results as studies with a response rate of 60%–70%. The questionnaire could be answered both via email and mobile phone, which was likely to be beneficial to OTNs. Some OTNs who had not studied nursing as a subject expressed concern, and some of them maybe chose not to answer. Others indicated ongoing organizational changes as a reason for not answering. The sample in this study was small and represented only the group that responded and not the entire population. Nevertheless, various factors have been identified that are important for the development of the OTNs’ clinical competence.

A questionnaire limits the answers one might receive, and therefore, we chose to supplement this with an open‐ended question where OTNs were given the opportunity to describe factors of importance for clinical competence development in perioperative nursing. The open‐ended question resulted in comprehensive descriptions and captured aspects that were not evident in the quantitative data, and thereby led to a deeper understanding of the research question (Creswell, 2014).

One strength of the present study was that clinically active OTNs throughout Sweden were invited to participate. There was also a variation of ages between 26 and 65 years, a wide range of professional experience, and OTNs employed in different hospitals. This might be seen as a representative sample of the population (Holbrook et al., 2007). The questionnaire chosen for the study has not been psychometrically tested in perioperative nursing but was pilot tested before the study started by OTNs working in different operating theatres in Sweden. Psychometric testing has begun, and the results are planned to be presented in an up‐coming study. The first and last authors performed the conventional content analysis of the open‐ended question. Quotes were used to strengthen the themes developed.

## 5 CONCLUSIONS

The study shows that the relationship between an academic degree and professional experience revealed that OTNs used different strategies for problem‐solving and taking responsibility for their decisions. Interprofessional learning and an academic degree are important for the OTNs’ clinical competence development. Academic clinical competence development is needed to ensure patient care in perioperative nursing. In addition, medical technology education should be supplemented with nursing care. More research is needed to validate the modified questionnaire PROFFSNurse SAS I in perioperative nursing. Research also needs to identify the need for further education in clinical competence and the influence of leadership in perioperative nursing.

## CONFLICT OF INTEREST

The authors declare that they do not have any conflicts of interest.

## References

[nop2352-bib-0001] Aiken, L. , Clarke, S. , Cheung, R. , Sloane, D. , & Silber, J. (2003). Educational levels of hospital nurses and surgical patient mortality. JAMA, 290(12), 1617–1623. 10.1001/jama.290.12.1617 14506121 PMC3077115

[nop2352-bib-0002] Aiken, L. , Sloane, D. , Bruyneel, L. , Van den Heede, K. , Griffiths, P. , Busse, R. , … Lesaffre, E. (2014). Nurse staffing and education and hospital mortality in nine European countries: A retrospective observational study. The Lancet, 383(9931), 1824–1830. 10.1016/S0140-6736(13)62631-8 PMC4035380 24581683

[nop2352-bib-0003] Aristoteles, (2012). Den nikomachiska etiken, 3rd ed Göteborg: Daidalos.

[nop2352-bib-0004] Bentling, S. , & Jonsson, B. (2010). Vårdpedagogiska utmaningar. Stockholm: Liber.

[nop2352-bib-0005] Blegeberg, B. , Blomberg, A. C. , & Hedelin, B. (2008). Nurses conceptions of the professional role of operation theatre and psychiatric nurses. Vård i Norden, 28(3), 9–13. https://doi.org/10:1177/010740830802800303

[nop2352-bib-0006] Blomberg, A‐C. , Bisholt, B. , & Lindwall, L. (2018a). Responsibility for patient care in perioperative practice. Nursing Open, 5(3), 414–421. 10.1002/nop2.153 30062035 PMC6056433

[nop2352-bib-0007] Blomberg, A.‐C. , Bisholt, B. , & Lindwall, L. (2018b). Value conflicts in perioperative practice. Nursing Ethics, 1–12. 10.1177/0969733018798169 30345880

[nop2352-bib-0008] Blomberg, A.‐C. , Bisholt, B. , Nilsson, J. , & Lindwall, L. (2014). Making the invisible visible ‐ operating theatre nurses perceptions of caring in perioperative practice. Scandinavian Journal of Caring Sciences, 29(2), 361–368. 10.1111/scs.12172 25250842

[nop2352-bib-0009] Boyle, D. K. , Cramer, E. , Potter, C. , Gatua, M. W. , & Stobinski, J. X. (2014). The relationship between direct‐care RN specialty certification and surgical patient outcomes. AORN Journal, 100(5), 511–528. 10.1016/j.aorn.2014.04.018 25443121

[nop2352-bib-0010] Bull, R. M. , & Fitzgerald, M. (2004). The invisible nurse: Behind the scenes in an Australian OR. AORN Journal, 79(4), 810–823. 10.1016/S001-2092(06)60822-3 15115352

[nop2352-bib-0011] Bull, R. M. , & FitzGerald, M. (2006). Nursing in a technological environment: Nursing care in the operating room. International Journal of Nursing Practice, 12(1), 3–7. 10.1111/j.1440-172X.2006.00542.x 16403190

[nop2352-bib-0012] Cowin, L. S. , Hengstberger‐Sims, C. , Eagar, S. C. , Gregory, L. , Andrew, S. , & Rolley, J. (2008). Competency measurements: Testing convergent validity for two measures. Journal of Advanced Nursing, 64(3), 272–277. 10.1111/j.1365-2648.2008.04774.x 18990106

[nop2352-bib-0013] Creswell, J. W. (2014). A concise introduction to mixed methods research. Los Angeles: Sage Publications.

[nop2352-bib-0014] Declaration of Helsinki (2013). Ethical Principles for Medical Research Involving Human Subjects. Retrieved from http://www.wma.net/en/30publications/10policies/b3/

[nop2352-bib-0015] Ellström, P.‐E. (1992). Kompetens, utbildning och lärande i arbetslivet: Problem, begrepp och teoretiska perspektiv. Stockholm: Publica.

[nop2352-bib-0016] Eriksson, K. , & Lindström, U. Å. (2003). Gryning II: Klinisk Vårdvetenskap. Vasa: Inst. för vårdvetenskap, Åbo Akademi.

[nop2352-bib-0017] Finnbakk, E. , Wangensteen, S. , Skovdahl, K. , & Fagerström, L. (2015). The Professional Nurse Self‐Assessment Scale: Psychometric testing in Norwegian long term and home care contexts. BMC Nursing, 14(1), 1–13. 10.1186/s12912-015-0109-3 26578847 PMC4647290

[nop2352-bib-0018] Gillespie, B. M. , Chaboyer, W. , Longbottom, P. , & Wallis, M. (2010). The impact of organisational and individual factors on team communication in surgery: A qualitative study. International Journal of Nursing Studies, 47(6), 732–741. 10.1016/j.ijnurstu.2009.11.001 19945107

[nop2352-bib-0019] Gillespie, B. M. , Chaboyer, W. , & Wallis, M. (2007). The influence of personal characteristics on the resilience of operating room nurses: A predictor study. International Journal of Nursing Studies, 46(7), 968–976. 10.1016/j.ijnursstu.2007.08.006 17915223

[nop2352-bib-0020] Gillespie, B. M. , Chaboyer, W. , Wallis, M. , & Werder, H. (2011). Education and experience make a difference: Results of a predictor study. AORN Journal, 94(1), 78–90. 10.1016/j.aorn.2010.11.037 21722773

[nop2352-bib-0021] Gillespie, B. M. , & Hamlin, L. (2009). A synthesis of the literature on “competence” as it applies to perioperative nursing. AORN Journal, 90(2), 245–258. 10.1016/j.aorn.2009.07.011 19664414

[nop2352-bib-0022] Gillespie, B. M. , Polit, D. F. , & Chaboyer, W. (2013). The influence of personal characteristics on perioperative nurses' perceived competence: Implications for workforce planning. Australian Journal of Advanced Nursing, 30(3), 14–25.

[nop2352-bib-0023] Holbrook, A. , Krosnik, J. , & Pfent, A. (2007). The causes and consequences of response rates in surveys by the news media and government contractor survey research firms In Lepkowski J., Tucker N., Brick J., DeLeeuw E., Japec L., & Lavrakas P. (Eds.), Advances in telephone survey methodology (pp. 499–528). New York: Wiley.

[nop2352-bib-0024] Hsieh, H.‐F. , & Shannon, S. E. (2005). Three approaches to qualitative content analysis. Qualitative Health Research, 15(9), 1277–1288. 10.1177/1049732305276687 16204405

[nop2352-bib-0025] International Council for Nurses (2013). Code of ethics for nurses. Retrieved from http://www.icn.ch/images/stories/documents/about/icncode_swedish.pdf

[nop2352-bib-0026] Jaensson, M. , Falk‐Brynhildsen, K. , Gillespie, B. M. , Wallentin, F. Y. , & Nilsson, U. (2018). Psychometric validation of the Perceived Perioperative Competence Scale‐Revised in the Swedish context. Journal of Perianesthesia Nursing, 33(4), 499–511. 10.1016/j.jopan.2016.09.012 30077294

[nop2352-bib-0027] Jerlock, M. , Falk, K. , & Severinsson, E. (2003). Academic nursing education guidelines: Tool for bridging the gap between theory, research and practice. Nursing & Health Sciences, 5(3), 219–228. 10.1046/j.1442-2018.2003.00156.x 12877723

[nop2352-bib-0028] Lindwall, L. , & von Post, I. (2000). Perioperativ vård: Den perioperativa vårdprocessen. Lund: Studentlitteratur.

[nop2352-bib-0029] Luck, E. S. , & Gillespie, B. M. (2017). Technological advancements in the OR: Do we need to redefine intraoperative nursing roles? AORN journal, 106(4), 280–282. 10.1016/j.aorn.2017.08.012 28958313

[nop2352-bib-0030] McGarvey, H. E. , Chambers, M. G. A. , & Boore, J. R. P. (2000). Development and definition of the role of the operating department nurse: A review. Journal of Advanced Nursing, 32(5), 1092–1100. 10.1046/j.1365-2648.2000.01578.x 11114993

[nop2352-bib-0031] Meretoja, R. , Isoaho, H. , & Leiono‐Kilpi, H. (2004). Nurse competence scale: Development and psychometric testing. Journal of Advanced Nursing, 47(2), 124–133. 10.1111/j.1365-2648.2004.03071.x 15196186

[nop2352-bib-0032] Meretoja, R. , Leino‐Kilpi, H. , & Kaira, A.‐M. (2004). Comparison of nurse competence in different hospital work environments. Journal of Nursing Management, 12(5), 329–336. 10.1111/j.1365-2834.2004.00422.x 15315489

[nop2352-bib-0033] Mitchell, L. , Flin, R. , Yule, S. , Mitchell, J. , Coutts, K. , & Youngson, G. (2011). Thinking ahead of the surgeon: An interview study to identify scrub nurses’ non‐technical skills. International Journal of Nursing Studies, 48(7), 818–828. 10.1016/j.ijnurstu.2010.11.005 21190685

[nop2352-bib-0034] Richardson-Tench, M. (2008). The scrub nurse: Basking in reflecting glory. Journal of Advanced Periperative Care, 3(4), 125–131.

[nop2352-bib-0035] Smith, J. , & Palesy, D. (2018). Technology stress in perioperative nursing: An ongoing concern. ACORN, 31(2), 25–28.

[nop2352-bib-0036] Spruce, L. (2013). Bringing back the basics of perioperative nursing care. AORN Journal, 98(5), 438–439. 10.1016/j.aorn.2013.09.001 24209792

[nop2352-bib-0037] Stobinski, J. X. (2015). Certification and patient safety. AORN Journal, 101(3), 374–378. 10.1016/j.aorn.2014.11.018 25707730

[nop2352-bib-0038] Sweeney, P. (2010). The effects of information technology on perioperative nursing. AORN Journal, 92(5), 528–543. 10.1016/j.aorn.2010.02.016 21040817

